# Anti-Osteogenic Effect of Danshensu in Ankylosing Spondylitis: An *in Vitro* Study Based on Integrated Network Pharmacology

**DOI:** 10.3389/fphar.2021.772190

**Published:** 2021-11-25

**Authors:** Jiaxiao Li, Zexin Chen, Hongbo Liao, Yanting Zhong, Junying Hua, Miaoling Su, Jiahao Li, Jinrong Xu, Liao Cui, Yang Cui

**Affiliations:** ^1^ School of Medicine, South China University of Technology, Guangzhou, China; ^2^ Department of Rheumatology and Immunology, Guangdong Provincial People’s Hospital, Guangdong Academy of Medical Sciences, Guangzhou, China; ^3^ Department of Rheumatology and Immunology, South China Hospital of Shenzhen University, Shenzhen, China; ^4^ Guangdong Provincial Key Laboratory of Research and Development of Natural Drugs, and School of Pharmacy, Guangdong Medical University, Dongguan, China; ^5^ Department of Cardiology, The Second Affiliated Hospital of Guangdong Medical University, Zhanjiang, China

**Keywords:** ankylosing spondylitis, Danshensu, network pharmacology, ossification, fibroblast, osteoblast

## Abstract

Ankylosing spondylitis (AS) is a chronic inflammatory disease characterized by abnormal bone metabolism, with few effective treatments available. Danshensu [3-(3,4-dihydroxy-phenyl) lactic acid) is a bioactive compound from traditional Chinese medicine with a variety of pharmacologic effects. In the present study, we investigated the pharmacologic effect and molecular mechanism of Danshensu in AS. Potential targets of Danshensu were identified in four drugs-genes databases; and potential pharmacologic target genes in AS were identified in three diseases-genes databases. Differentially expressed genes related to AS were obtained from the Gene Expression Omnibus database. Overlapping targets of Danshensu and AS were determined and a disease–active ingredient–target interaction network was constructed with Cytoscape software. Enrichment analyses of the common targets were performed using Bioconductor. To test the validity of the constructed network, an *in vitro* model was established by treating osteoblasts from newborn rats with low concentrations of tumor necrosis factor (TNF)-α. Then, the *in vitro* model and AS fibroblasts were treated with Danshensu (1–10 μM). Osteogenesis was evaluated by alkaline phosphatase staining and activity assay, alizarin red staining, quantitative PCR, and western blotting. We identified 2944 AS-related genes and 406 Danshensu targets, including 47 that were common to both datasets. The main signaling pathways associated with the targets were the c-Jun N-terminal kinase (JNK) and extracellular signal-regulated kinase (ERK) pathways. A low concentration of TNF-α (0.01 ng/ml) promoted the differentiation of osteoblasts; this was inhibited by Danshensu, which had the same effect on AS fibroblasts but had the opposite effect on normal osteoblasts. Danshensu also decreased the phosphorylation of JNK and ERK in AS fibroblasts. There results provide evidence that Danshensu exerts an anti-osteogenic effect *via* suppression of JNK and ERK signaling, highlighting its therapeutic potential for the treatment of AS.

## Introduction

Ankylosing spondylitis (AS) is a chronic progressive inflammatory disease with insidious onset characterized by chronic inflammatory back pain, bone resorption, and new bone formation, with the latter leading to ankylosis and functional disability (Poddubnyy and Sieper, 2017; Lories, 2018). The pathogenesis of AS and therapeutic strategies targeting ectopic new bone formation have been the focus of many studies (Qin et al., 2018; van Tok et al., 2019). Heterotopic ossification involves endochondral and membranous bone formation and cartilage metaplasia, in which fibroblasts and bone cells such as mesenchymal stem cells, osteoblasts, and osteoclasts play an essential role (Van Mechelen et al., 2018; Liu et al., 2021). Inflammatory cytokines such as tumor necrosis factor (TNF)-α, interleukin (IL)-17A, and IL-6 are also implicated in new bone formation (Li et al., 2016; van Tok et al., 2019; Zou et al., 2020). TNF-α is known to inhibit osteoblast differentiation but at low concentrations, it not only enhanced proliferation but also promoted osteoblast differentiation in AS *via* Wnt signaling and subsequent bone formation *via* nuclear factor (NF)-κB (p65) and c-Jun N-terminal kinase (JNK)/activator protein (AP)-1 signaling (Frost et al., 1997; Li et al., 2018). Besides, low concentration of TNF-α enhanced the osteogenic differentiation of AS fibroblasts, which was accompanied by the expression of osteogenesis markers including Runt-related transcription factor (RUNX)2, osteopontin (OPN), and osteocalcin (OCN) (Zou et al., 2020).

As advanced ankylosis of the spine is the worst long-term outcome of AS, preventing ectopic new bone formation is a major focus of research. However, there are few drugs available that prevent ankylosis progression (Poddubnyy and Sieper, 2020). Biologic agents targeting TNF-α and IL-17A are important treatments that alleviate AS symptoms by suppressing inflammation and slowing spinal radiographic progression. However, not all patients respond to these treatments, which can increase the risk of infection; moreover, the high cost of these agents imposes a considerable economic burden on patients (Yin et al., 2020). The therapeutic effect of TNF-α blockers in new bone formation of AS still leave a question open. It has been shown to suppress inflammation but also enhance radiographic progression in some studies (Maksymowych et al., 2009; Pedersen et al., 2011). Therefore, there is an urgent need for alternative, effective medicines for the treatment of AS.

Danshensu [3-(3,4-dihydroxy-phenyl) lactic acid], is a bioactive compound from traditional Chinese medicine--*Salvia miltiorrhiza* (Danshen) with a variety of pharmacologic effects (Zeng et al., 2017; Wang et al., 2021; Xie et al., 2021). Danshensu has been integrated into the treatment of cardiovascular diseases (e.g., myocardial ischemia and reperfusion, atherosclerosis, and hypertension) and cerebrovascular diseases (e.g., cerebral embolism) owing to its capacity to modulate inflammation and exert antioxidative and anti-apoptotic effects (Zhang et al., 2019). Danshensu was shown to suppress neuroinflammation by reducing the levels of proinflammatory cytokines such as TNF-α and IL-6 in the hippocampus (Zhang et al., 2019), and exerted a protective effect against myocardial ischemia/reperfusion injury by blocking the phosphorylation and activation of JNK and nuclear translocation of nuclear factor (NF)-κB, which is involved in new bone formation in AS (Meng et al., 2016; Li et al., 2018). Danshensu plays an important role in bone physiology by regulating osteoblast differentiation and function. An H_2_S-releasing compound derived from Danshensu was found to have an antioxidant effect in an H_2_O_2_-induced cell injury model in MC3T3-E1 osteoblasts by attenuating the activation of the p38 mitogen-activated protein kinase (MAPK), extracellular signal-regulated kinase (ERK)1/2, and JNK–MAPK pathways (Yan et al., 2017). On the contrary, in our previous study we found that salvianolic acid B, another bioactive component of Danshen, promoted osteogenesis of normal human mesenchymal stem cells (hMSCs) by enhancing ERK1/2 signaling (Xu et al., 2014). However, little is known about the application or effect of Danshensu in AS.

**FIGURE 1:**
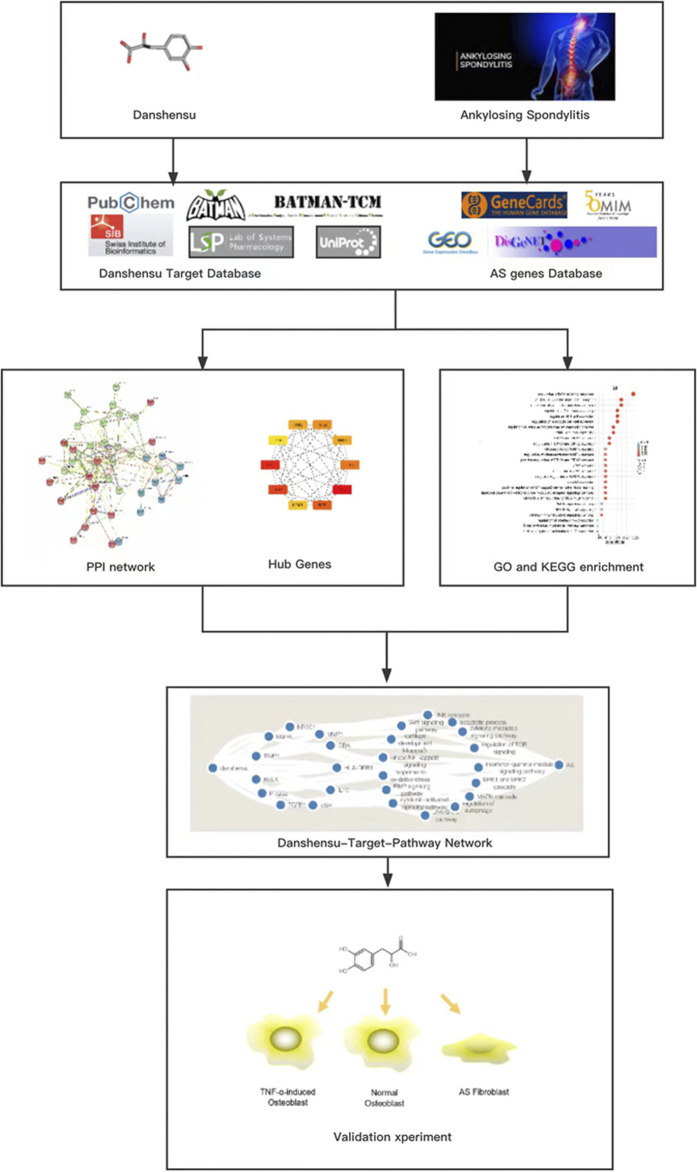
Workflow of systems pharmacology analysis and experimental assessment.

Based on the above findings, we speculated that Danshensu can prevent ossification of osteoblasts and fibroblasts in AS. To test this hypothesis, in this study we investigated the effect and mechanism of action of Danshensu on osteoblast differentiation in AS. We first conducted a network pharmacology analysis to identify potential targets of Danshensu and analyzed the function and pathways related to the targets. We then constructed a disease–drug component–target–signaling pathway interaction network to identify important pathways, and evaluated the efficacy and mechanism of action of Danshensu using osteoblasts and AS fibroblasts (Figure 1).

## Materials and Methods

### Databases and Software

The following databases were used in the network pharmacology analysis: Bioinformatics Analysis Tool for Molecular Mechanism of Traditional Chinese Medicine (BATMAN-TCM; http://bionet.ncpsb.org.cn/batman-tcm/); Traditional Chinese Medicine Systems Pharmacology (TCMSP; https://tcmsp-e.com/), SwissTargetPrediciton (http://swisstargetprediction.ch/); Pharmmapper (http://lilab-ecust.cn/pharmmapper/index.html); GeneCards (https://www.genecards.org/), PubChem (https://pubchem.ncbi.nlm.nih.gov/); Online Mendelian Inheritance in Man (OMIM; https://www.omim.org/); DisGeNET (https://www.disgenet.org/home/); GeneCards (https://www.genecards.org/); Gene Expression Omnibus (GEO; https://www.ncbi.nlm.nih.gov/geo/), UniProt (https://www.uniprot.org/); Kyoto Encyclopedia of Genes and Genomes (KEGG; https://www.genome.jp/kegg/); and Search Tool for the Retrieval of Interacting Genes/Proteins (STRING; https://string-db.org/). The following software programs were used: Cytoscape v3.8.2 (https://cytoscape.org/); Rx64 v4.1.0 (https://cran.r-project.org/); and Biocondcutor (https://www.bioconductor.org/).

### Danshensu Target Identification

The Simplified Molecular Input Line Entry System (SMILES) and International Chemical Identifier key (InChiKey) for Danshensu were retrieved from the PubChem database. Danshensu was searched in BATMAN-TCP (prediction score of the target gene ≥20; adjusted *p* value of the prediction result <0.05), TCMSP, SwissTargetPrediction, and Pharmamapper databases based on the results of the search.

### Identification of Pharmacologic Targets in AS

The keyword “ankylosing spondylitis” was used as input in the OMIM, GeneCards, and DisGeNET databases to identify genes related to AS. The original microarray data of GSE25101 and GSE73754 were analyzed with GEO2R to identify differentially expressed genes (DEGs) and generate a volcano plot; according to log_2_ fold change (FC), the −log_10_ (adjusted *p* values) were divided into upregulated genome, downregulated genome, no statistical difference, and non-expressed difference genome (screening conditions: adjusted *p* value <0.01, |log_2_ FC|≥1.0). Both AS-related gene and DEG names were normalized and merged and duplicate items were deleted, and the obtained gene sets were used as disease targets.

### Standardization and Screening for Potential AS Targets of Danshensu

All targets were searched in the UniProt database with the species set to human, and the collected protein or gene information was corrected to the standard UniProt name (Symbol) of the target. The set of Danshensu targets was mapped to the set of disease targets and intersected to obtain the potential targets of Danshensu in AS.

### Identification of Hub Genes Shared Between Danshensu Target and AS Genes

Topologic properties, degree, and interaction of the distribution network from the STRING database were analyzed using the cytoHubba plugin of Cytoscape.

### Gene Ontology Functional Annotation and KEGG Pathway Enrichment Analysis

The drug component–disease target gene names were encoded by UniProt IDs. GO functional annotation of the targets and KEGG pathway enrichment analysis were performed using Rx64 v4.1.0 software with bioc-Lite.R, clusterProfliter.R, and pathview.R packages from the Bioconductor platform.

### Construction of Disease–Drug Component–Target–Signaling Pathway Interaction Network

Cytoscape v3.8.2 software was used to construct the disease–drug component–target–signaling pathway interaction network, in which the nodes represented the disease, drug component, target, and signaling pathways and edges represented interactions between drug component and target, target and AS, or target and signaling pathway. The topologic properties of the network were analyzed using the Network Analysis plugin of Cytoscape.

### Cell Culture

Hip capsular ligaments were collected from three AS patients at Guangdong Provincial People’s Hospital. The study was approved by the ethics committee of the hospital. Fibroblasts were extracted from the hip capsular ligaments as previously described (Qin et al., 2018). Briefly, ligaments were washed twice with phosphate-buffered saline (PBS) and then cut into small pieces that were placed in 10-mm culture dishes. After 3–4 h, Dulbecco’s Modified Eagle’s Medium (DMEM; Gibco, Shanghai, China) containing 15% fetal bovine serum (FBS; Gibco) and 1% penicillin–streptomycin (Solarbio, Beijing, China) was added to the culture dishes. After cells had adhered, the ligament pieces were removed and the medium was replaced with fresh medium containing 10% FBS and 1% penicillin–streptomycin. The medium was changed every 3–4 days and cells from passage 3–5 were used for experiments.

Primary osteoblasts from the skull of 24-h newborn Sprague–Dawley rats were provided by Guangdong Medical University. Briefly, the calvaria was cut into small pieces and digested at 37°C with trypsin for 15 min, followed by 0.1% collagenase I for 20 min. The supernatant was centrifuged at 800 rpm for 10 min, and the cell precipitate was collected and inoculated in 25-cm^2^ culture vials while the supernatant was transferred back to the centrifuge tube containing the bone fragments for additional digestion. The above steps were repeated 3 times. The cells were grown in DMEM containing 10% FBS and 1% penicillin–streptomycin at 37°C in a humidified atmosphere of 5% CO_2_. The culture medium was refreshed every 2–3 days. When the cells reach 80% confluence, they were digested with 0.25% EDTA–trypsin (Solarbio) and passaged in a 1:2 or 1:3 ratio. Cells from passage 3–5 were used for experiments.

### Cell Proliferation Assay

The 3-(4,5-dimethylthiazol-2-yl)-2,5-diphenyltetrazolium bromide (MTT) assay was used to evaluate the effect of Danshensu on osteoblast and AS fibroblast proliferation. The cells were seeded in 96-well plates at a density of 3×10^3^ (osteoblasts) and 5×10^3^/well (fibroblasts), respectively. After 24 h, the cells were divided into nine groups (5 wells per group) that were treated with Danshensu at a concentration of 0, 0.1, 0.3, 1, 3, 10, 30, 100, or 300 μM. After incubation for 7 days, 20 μl MTT (5 mg/ml) was added to each well. The mixed medium was discarded 4 h later and replaced with 150 μl dimethylsulfoxide (Solarbio), and the plate was agitated for 20 min at room temperature. A microplate reader (Bio-Rad Laboratories, Hercules, CA, United States) was used to measure the optical density (OD) of each well at a wavelength of 570 nm.

### Alkaline Phosphatase Staining and Activity Assay

Cells were washed twice with PBS and fixed with 4% paraformaldehyde for 30 min. After three washes with PBS, the cells were stained with ALP detection solution for 30 min at room temperature according to instructions of the ALP kit (Leagene, Beijing, China). To measure ALP activity, cells were lysed overnight at 4°C with radioimmunoprecipitation assay (RIPA) lysis buffer (Beyotime, Shanghai, China), followed by incubation for 15 min at 37°C in ALP detection buffer from the ALP activity kit (Jiancheng, Nanjing, China). The reaction was terminated and coloring solution was added to each well. The OD was measured at 520 nm with a microplate reader. The protein concentration of each well was measured with a bicinchoninic acid protein assay kit (Beyotime). ALP activity is reported as U/100 g of protein.

### Alizarin Red Staining

Cells were washed twice with PBS and then fixed with 70% ethanol for 20 min at room temperature; they were then washed with water and stained with 0.2% alizarin red (Sigma, Tokyo, Japan) solution (pH 4.1) for 30 min. The solution was discarded and the cells were washed 3 times with distilled water.

### RNA Isolation and Quantitative (q)PCR

After culturing for 14 days, total RNA was extracted from cells grown in 6-wells plates using TRIzol reagent (Ambion, Austin, TX, United States). The RNA was reverse transcribed using the PrimeScript RT reagent kit (Takara, Dalian, China) and qPCR was carried out to detect mRNA levels of Runt-related transcription factor (RUNX)2, osterix (SP7), osteocalcin (OCN), bone morphogenetic protein (BMP)2, and glyceraldehyde 3-phosphate dehydrogenase (GAPDH) (internal control). Cycling conditions were as follows: 95°C for 5 min, followed by 40 cycles of 95°C for 15 s and 60°C for 60 s. Forward and reverse primers were synthesized by Sangon Biotech (Shanghai, China) and are shown in Table 1.

**TABLE 1:** Primer sequences of genes related to ankylosing spondylitis.

Gene	Species	Forward	Reverse
OCN	Human	5′-AGG​GCA​GCG​AGG​TAG​TGA​AGA​G-3′	5′-GCC​GAT​GTG​GTC​AGC​CAA​CTC-3′
SP 7	Human	5′-CGG​CAA​GAG​GTT​CAC​TCG​TTC​G-3′	5′-TGG​AGC​AGA​GCA​GGC​AGG​TG-3′
RUNX 2	Human	5′-AGG​CAG​TTC​CCA​AGC​ATT​TCA​TCC-3′	5′-TGG​CAG​GTA​GGT​GTG​GTA​GTG​AG-3′
GAPDH	Human	5′- CAG​GAG​GCA​TTG​CTG​ATG​AT-3′	5′- GAAGGCTGGGGCTCATTT -3′
*BMP 2*	Rat	5′-AAG​CGT​CAA​GCC​AAA​CAC​AAA​CAG-3′	5′-CCA​GTC​ATT​CCA​CCC​CAC​ATC​AC-3′
*RUNX 2*	Rat	5′-TCC​GCC​ACC​ACT​CAC​TAC​CAC-3′	5′-GAA​CTG​ATA​GGA​CGC​TGA​CGA​AG-3′
*SP 7*	Rat	5′-GCC​TAC​TTA​CCC​GTC​TGA​CTT​TGC-3′	5′-CCC​TCC​AGT​TGC​CCA​CTA​TTG​C-3′
*GAPDH*	Rat	5′- GAC​ATG​CCG​CCT​GGA​GAA​AC -3′	5′- AGC​CCA​GGA​TGC​CCT​TTA​GT -3′

### Western Blotting

After culturing for 14 days, cells were lysed with RIPA lysis buffer supplemented with phenylmethylsulfonyl fluoride (Beyotime), and 30 μg of protein per well were separated by sodium dodecyl sulfate polyacrylamide gel electrophoresis on a 10% acrylamide gel and electrotransferred to a polyvinylidene difluoride membrane that was incubated with antibodies against GAPDH (1:2000; Abway, Shanghai, China); RUNX2 (1:1000), collagen type I (COL1; 1:1000), and SP7 (1:1000) (all from Abcam); OCN (1:500; Affinity, Cincinnatin, United States); and JNK (1:1000) and extracellular signal-regulated kinase (ERK; 1:1000) (both from Signalway Antibody, College Park, MD, United States). Enhanced chemiluminescence substrate (Beyotime) was used to detect the protein signal.

### Statistical Analysis

All data are presented as mean ± SD. SPSS v22.0 software was used to analyze the data. One-way analysis of variance was used to assess differences between groups, with a least significant difference post hoc test when groups had equal variance (*p* > 0.05) and Dunnett’s post hoc test in the alternative case. Differences with a *p* value < 0.05 were considered statistically significant.

## Results

### Identification of Danshensu Targets

**FIGURE 2:**
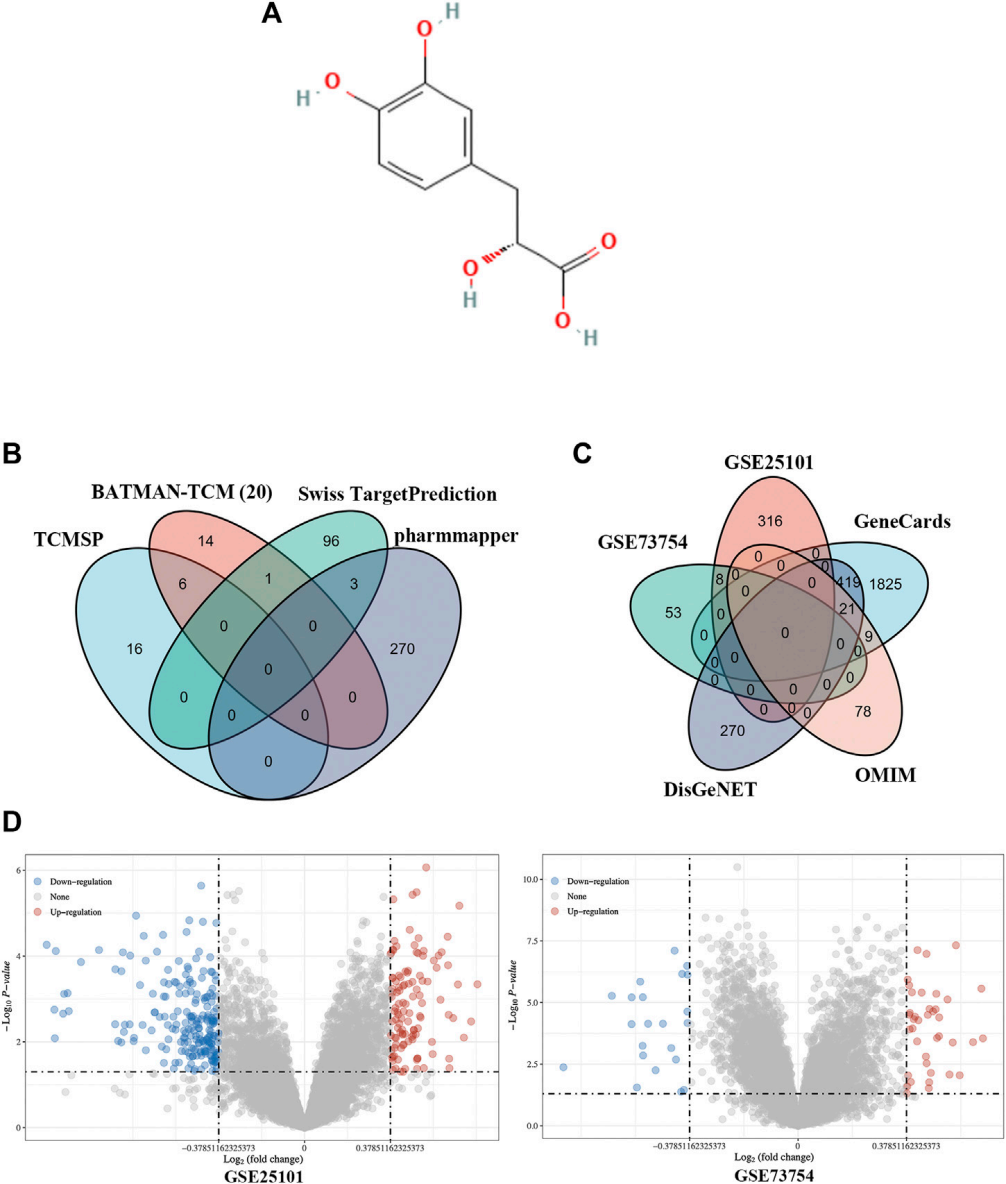
Identification of Danshensu targets and AS-related genes. **(A)** Chemical structure of Danshensu. **(B, C)** Venn diagram of Danshensu targets **(B)** and AS-related genes **(C)**. **(D)** Volcano plot of DEGs obtained from GSE25101 and GSE73754.

We identified 21 putative targets of Danshensu (Figure 2A) in BATMAN-TCM along with 22 in TCMSP, 100 in SwissTargetPrediction, and 273 in Pharmmapper (Figure 2B). The identified genes were aggregated and duplicates and inconsistently named genes were removed, leaving 406 genes as potential targets.

### Identification of AS-Related Genes

Using the keyword “ankylosing spondylitis,” 108 AS-related genes were identified in OMIM along with 2,274 in GeneCards and 710 in DisGeNET. A total of 324 and 61 DEGs were obtained from GSE25101 and GSE73754, respectively (Figure 2D). The identified genes were aggregated and duplicates and inconsistently named genes were removed, leaving 2,944 AS-related genes (Figure 2C).

### Screening and Protein–Protein Interaction Network Construction of Danshensu AS Targets

**FIGURE 3:**
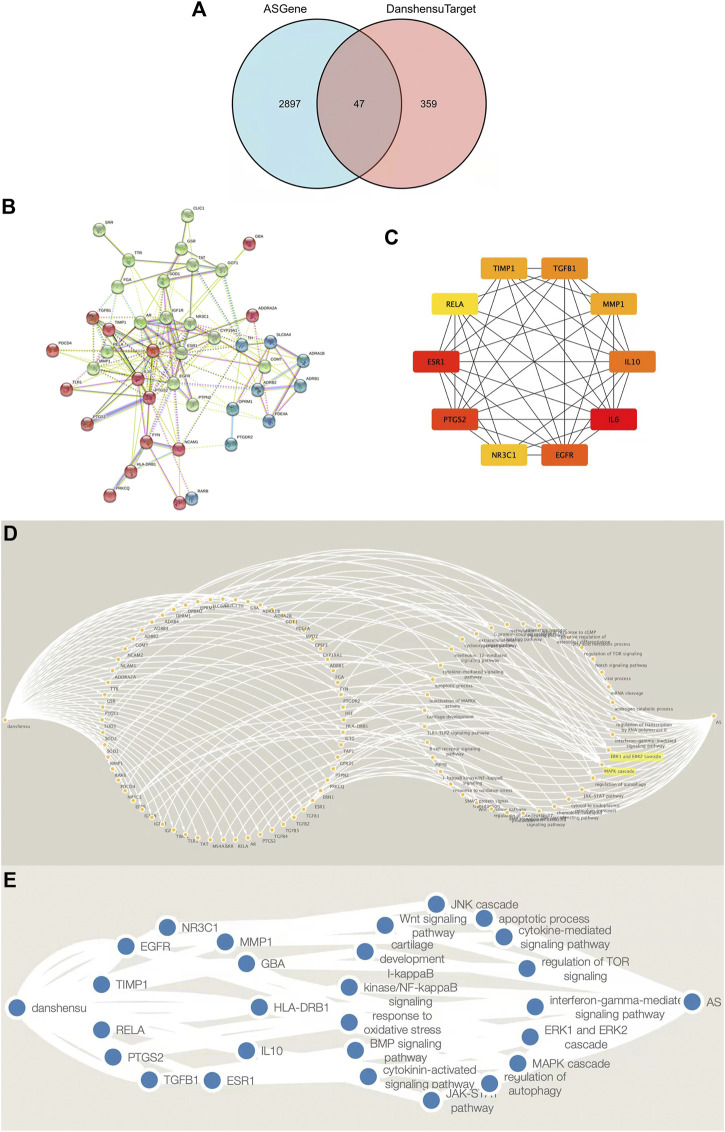
Screening and PPI network construction and Target-signaling pathway interaction network of Danshensu anti-AS targets. **(A)** Venn diagram of target genes of Danshensu in AS. **(B)** PPI network of Danshensu targets in AS. **(C)** Venn diagram of hub genes of Danshensu in AS. **(D)** Target–signaling pathway interaction network of Danshensu in AS. **(E)** Target-signaling pathway interaction network of hub genes in Danshensu against AS. Note: The left single node was Danshensu, the right single node was AS, the left nodes in circular arrangement were overlapping Danshensu-AS targets, and the left nodes in circular arrangement were signaling pathways.

AS-related genes and Danshensu targets were intersected in a Venn diagram, which yielded 47 drug component–disease targets (Figure 3A). The 47 genes were imported into the STRING database; the functions of multiple proteins were selected and a combined score >0.4 was used as the threshold while disregarding isolated nodes to obtain a PPI network for Danshensu AS targets. The topologic analysis of the network revealed 164 connections among 47 targets (Figure 3B).

### Identification and Verification of Hub Genes Among Danshensu Targets and AS Genes

Cytoscape was used to visualize key genes as a network from which overlapping genes were selected for further analysis by determining the node degree or interaction of sequenced candidate genes using the cytoHubba application. The top 10 hub genes in the network of Danshensu AS targets were *IL6*, *ESR1*, *PTGS2*, *EGFR*, *IL10*, *TGFB1*, *TIMP1*, *MMP1*, *NR3C1*, and *RELA* (Table 2 and Figure 3C).

**TABLE 2:** Top 10 genes in the protein–protein interaction network ranked by the maximal clique centrality method.

Rank	Name	Score
1	IL6	9,683
2	ESR1	9,608
3	PTGS2	9,588
4	EGFR	9,509
5	IL10	7,270
6	TGFB1	5,880
7	TIMP1	5,040
7	MMP1	5,040
9	NR3C1	3,020
10	RELA	2,185

### Drug Component–Target GO Function Annotation and KEGG Pathway Enrichment Analysis

**FIGURE 4:**
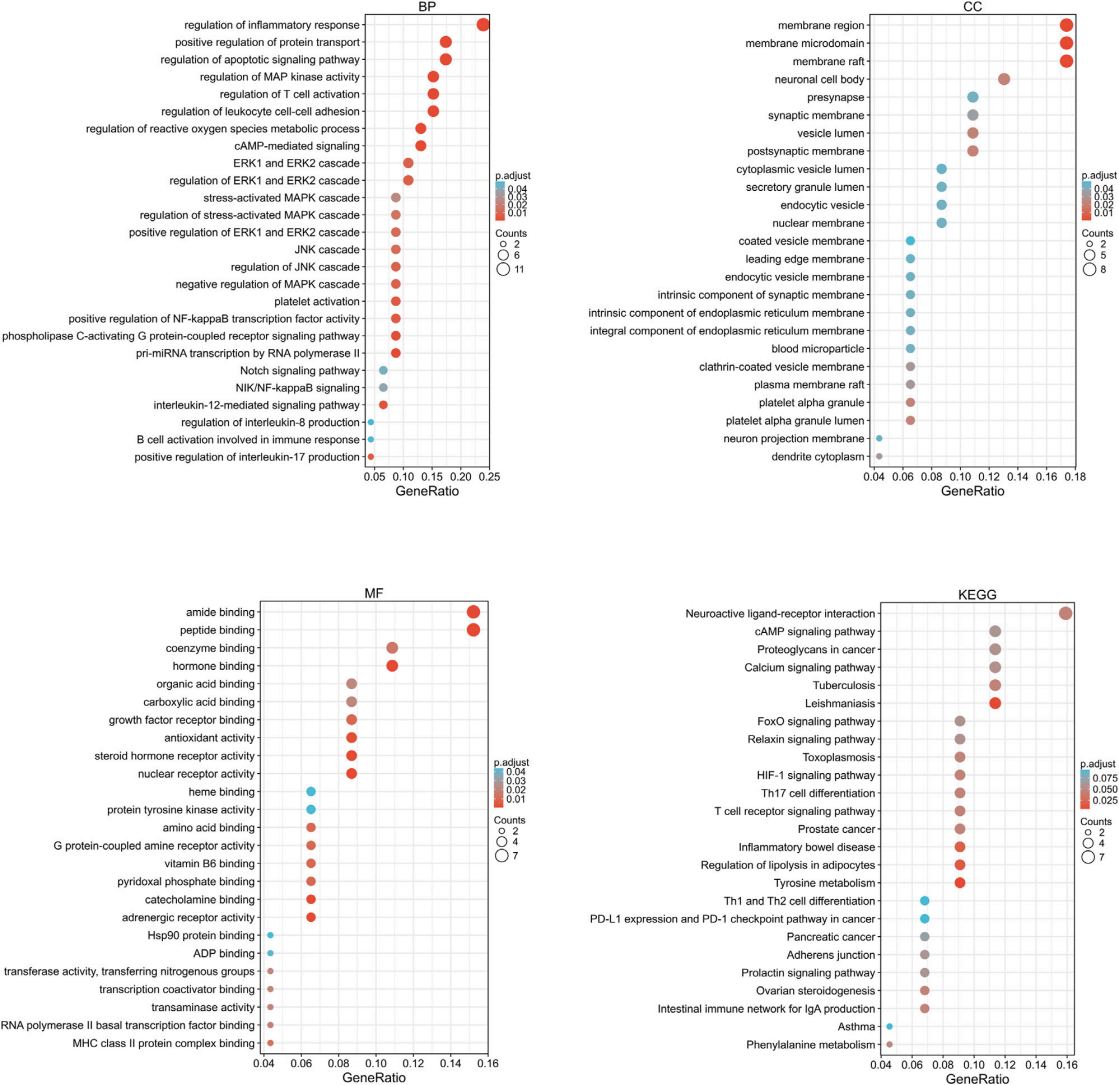
Drug component–target GO function annotation and KEGG pathway enrichment analysis. BP, biological process; CC, cellular component; MF, molecular function.

The target gene names (symbol) were transformed into gene IDs by searching the UniProt database, and the biocLite.R package of Bioconductor was used to perform GO functional annotation and KEGG pathway enrichment analysis of the target genes using Rx 64 v4.0.1 software, with *p* = 0.05 and Q = 0.05 as the threshold values. The top 40 GO terms are shown in Figure 4. Most were related to inflammatory response, oxidative stress, the MAPK cascade, etc. The KEGG pathway enrichment analysis yielded 13 signaling pathways among which ERK and JNK signaling pathways were highly enriched, suggesting that they are key pathways for the anti-osteogenic effect of Danshensu (Figure 4).

### Construction of a Disease–Drug Component–Target–Signaling Pathway Interaction Network

The drug component–disease target, active ingredient molecular ID, and drug component–disease–signaling pathway interaction data were imported into Cytoscape, and a drug component–target interaction network was generated using the Merge function in the software (Figures 3D,E). These targets were presumed to be key targets of Danshensu in AS.

### Danshensu has Little Effect on Osteoblast and AS Fibroblast Proliferation

**FIGURE 5:**
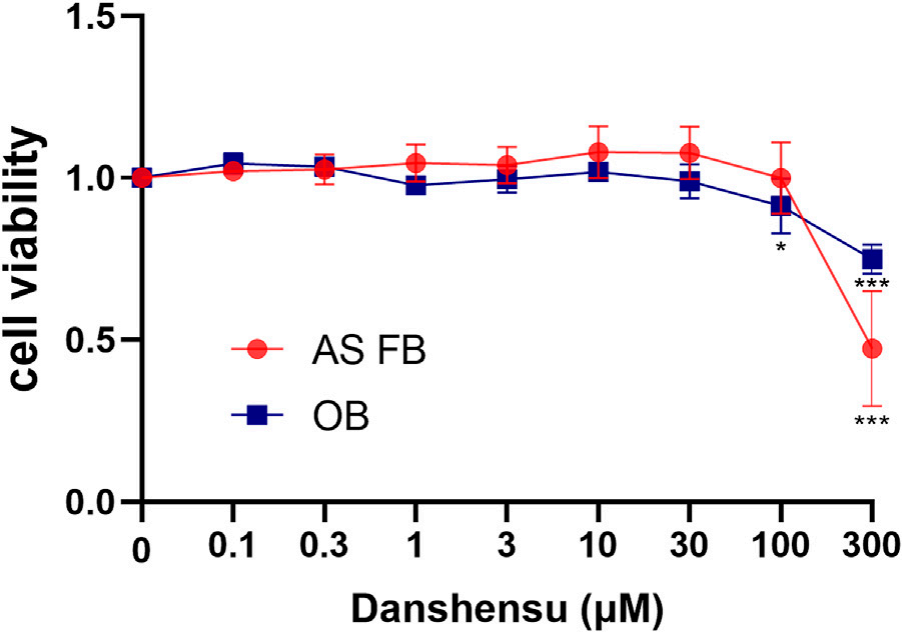
Viability of osteoblasts and AS fibroblasts treated with Danshensu at different concentrations.

To investigate the effect of Danshensu on osteoblast and AS fibroblast proliferation, the cells were exposed to increasing concentrations of Danshensu ranging from 0.1 to 300 μM. After culturing for 7 days, the MTT assay was performed. Danshensu did not affect the proliferation of osteoblasts and AS fibroblasts at concentrations between 0.1 and 30 μM, while at 100 μM osteoblast proliferation was significantly inhibited and at 300 μM, both osteoblast and AS fibroblasts were affected (Figure 5). We therefore used 1, 3, and 10 μM Danshensu for the following experiment.

### Low Concentrations of TNF-α Promote Osteoblast Differentiation

**FIGURE 6:**
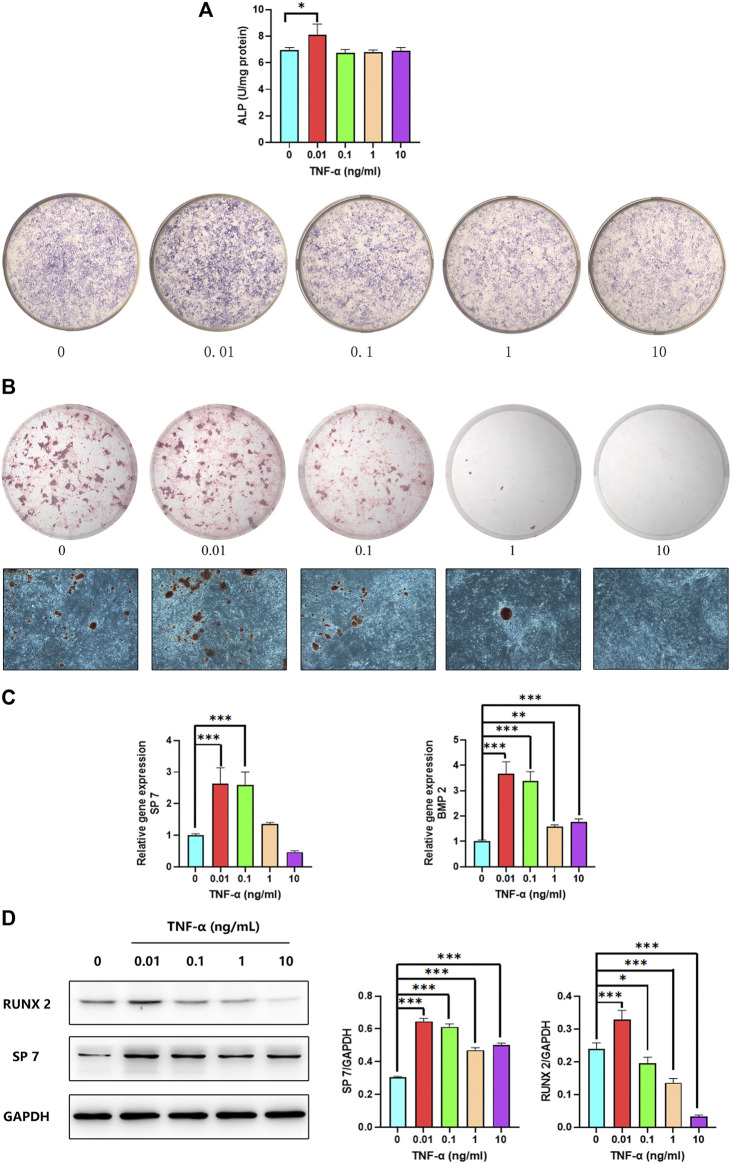
Low concentrations of TNF-α promote osteoblast differentiation. **(A)** ALP activity and staining of osteoblasts after 7 days of treatment with different concentrations of TNF-α. **(B)** ARS of osteoblasts on day 30. **(C)** *SP7* and *BMP2* mRNA expression in osteoblasts on day 14. **(D)** *RUNX2* and *SP7* protein levels on day 14. *p < 0.05, **p < 0.01, ***p < 0.001 vs. 0 group, *n* = 3.

We investigated the effect of TNF-α on osteoblast differentiation by ALP staining and activity assay after 7 days of treatment. Both ALP staining and activity were increased in osteoblasts by treatment with 0.01 ng/ml TNF-α for 7 days (Figure 6A). We also examined the effect of TNF-α on mineralization in osteoblasts by ARS. There were more calcium nodules in the group treated with 0.01 ng/ml TNF-α than in the other groups (Figure 6B). TNF-α at concentrations 1 and 10 ng/ml was not toxic to osteoblasts, and there were no calcium nodules in the 10 ng/ml group.

To confirm the effect of low TNF-α concentrations on osteoblast differentiation, we evaluated the expression of *BMP2* and *SP7*—2 genes related to this process—by qPCR. Application of 0.01 ng/ml TNF-α resulted in the upregulation of *BMP2* and *SP7* (Figure 6C). We also examined the levels of *RUNX2* and *COL1A1* proteins by western blotting and found that they were significantly increased in the presence of 0.01 ng/ml TNF-α, whereas a concentration of 10 ng/ml reduced the protein level of *RUNX2* (Figure 6D). Based on these results, 0.01 ng/ml TNF-α was used in subsequent experiments to create a proinflammatory environment for inducing osteoblast differentiation.

### Danshensu Inhibits TNF-α–Induced Osteogenic Differentiation

**FIGURE 7:**
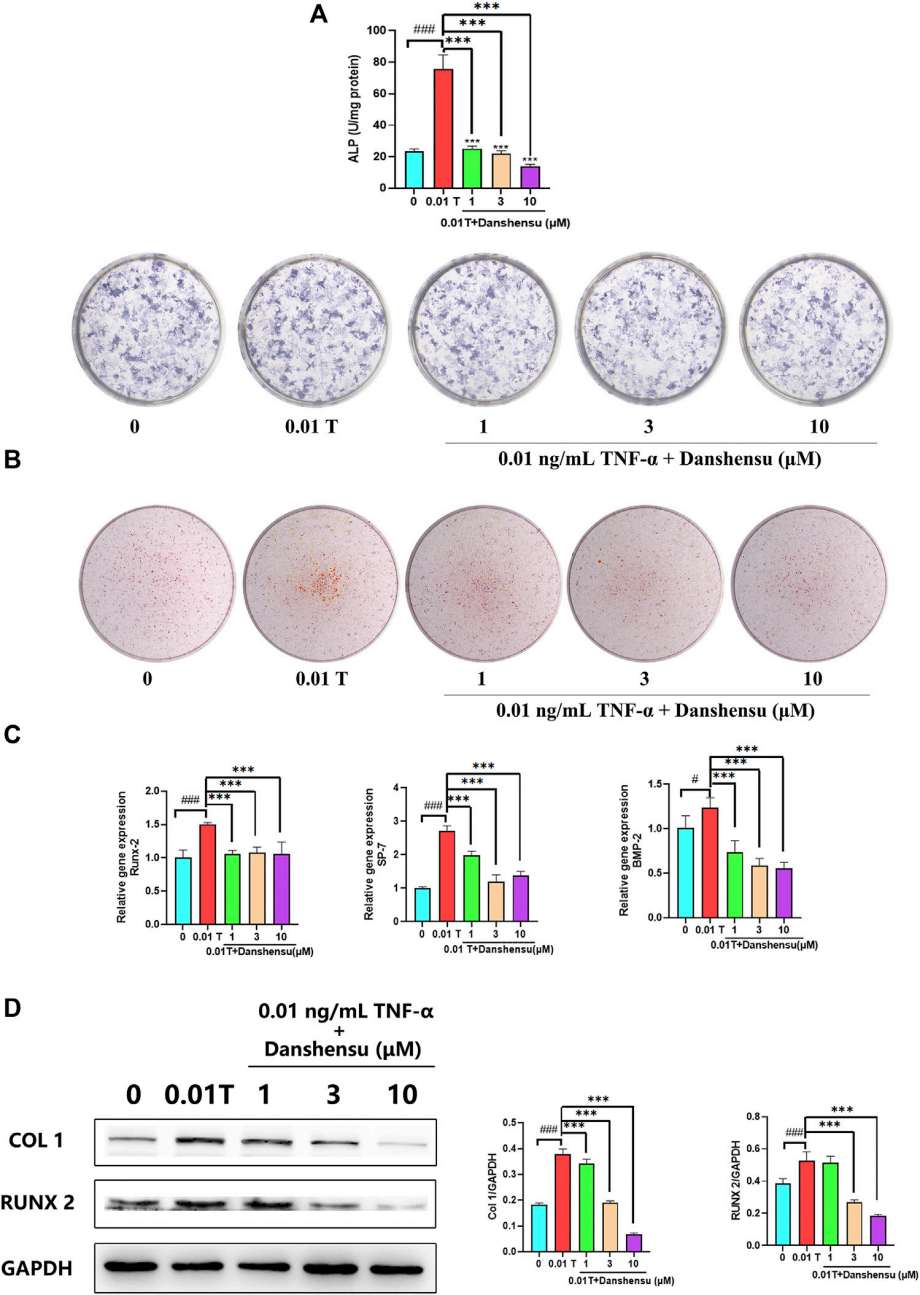
Danshensu inhibits TNF-α–induced osteogenic differentiation. **(A)** ALP activity and staining of osteoblasts after 7 days of treatment with TNF-α. **(B)** ARS of osteoblasts at days 30. **(C)** *SP7*, *BMP2*, and *RUNX2* mRNA expression in osteoblasts on day 14. **(D)** *RUNX2* and *COL1* protein levels on day 14. ^#^ *p* < 0.05, ^###^ *p* < 0.01 vs. 0 group; *p < 0.05, **p < 0.01, ***p < 0.001 vs. 0.01 T group, *n* = 3.

To investigate the anti-osteogenic effect of Danshensu, osteoblasts were treated with 0.01 ng/ml TNF-α and different concentrations of Danshensu, and ALP staining and activity were evaluated 7 days later. Danshensu inhibited ALP activity in TNF-α–induced osteoblasts in a dose-dependent manner. ALP staining was also reduced in cells treated with Danshensu compared to those treated with 0.01 ng/ml TNF-α only (Figure 7A). Similar results were obtained by ARS (Figure 7B). Additionally, qPCR analysis showed that *RUNX2*, *BMP2*, and *SP7* mRNA expression was reduced in cells treated with Danshensu compared to those treated with TNF-α only (Figure 7C) and after 14 days, cells exposed to 0.01 ng/ml TNF-α and 1, 3, or 10 μM Danshensu had lower levels of *COL1A1* and *RUNX2* proteins compared to those treated with TNF-α only (Figure 7D).

### Danshensu Promotes Ossification of Normal Osteoblasts

**FIGURE 8:**
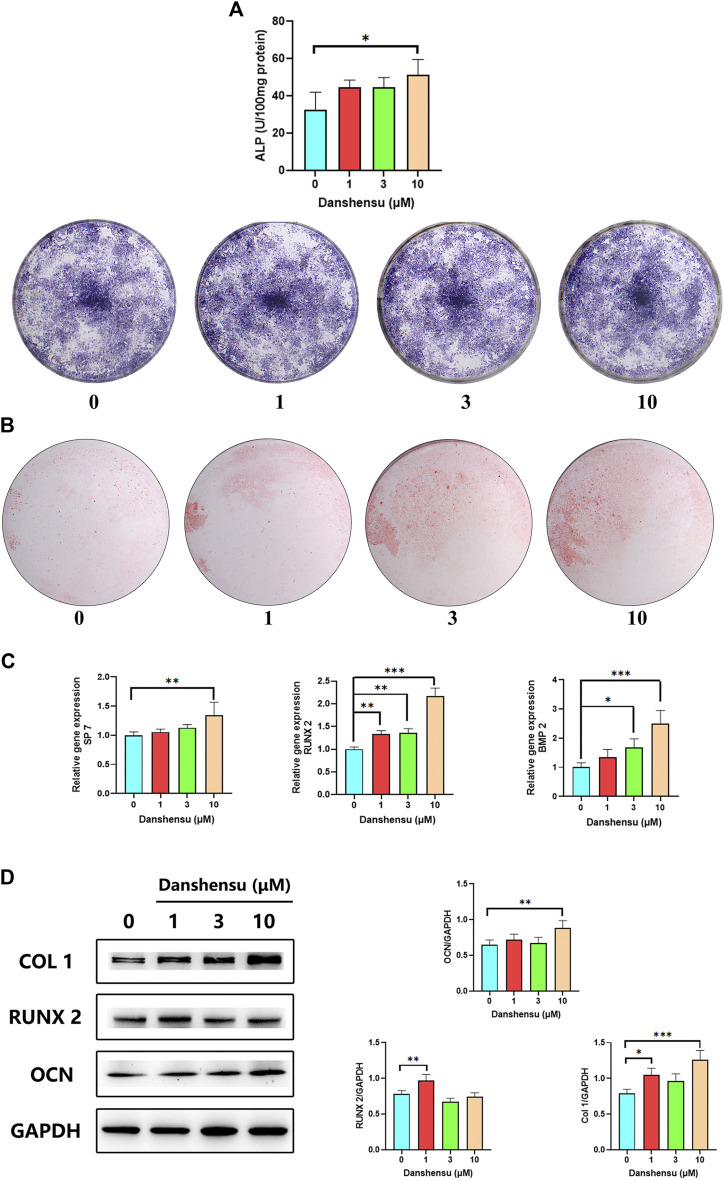
Danshensu promotes ossification of normal osteoblasts. **(A)** ALP activity and staining of osteoblasts after 7 days of treatment with different concentrations of Danshensu. **(B)** ARS of osteoblasts on day 30. **(C)** *SP7*, *BMP2*, and *RUNX2* mRNA expression in osteoblasts on days 14. **(D)** *RUNX2*, *COL1*, *OCN*, and *GAPDH* protein levels on days 14. *p < 0.05, **p < 0.01, ***p < 0.001 vs. 0 group, *n* = 3.

To determine whether Danshensu affects normal osteoblasts, we assessed the osteogenic potential of osteoblasts treated with 1, 3, and 10 μM of Danshensu. The results of ALP staining and the activity assay showed that Danshensu increased osteogenic differentiation (Figure 8A); this was confirmed by ARS (Figure 8B). The qPCR analysis showed that *RUNX2*, *BMP2*, and *SP7* mRNA expression was increased by Danshensu treatment, an effect that was concentration-dependent (Figure 8C); and a similar effect was observed on *COL1A1* and *RUNX2* protein levels (Figure 8D).

### Danshensu Suppresses Ossification of AS Fibroblasts

**FIGURE 9:**
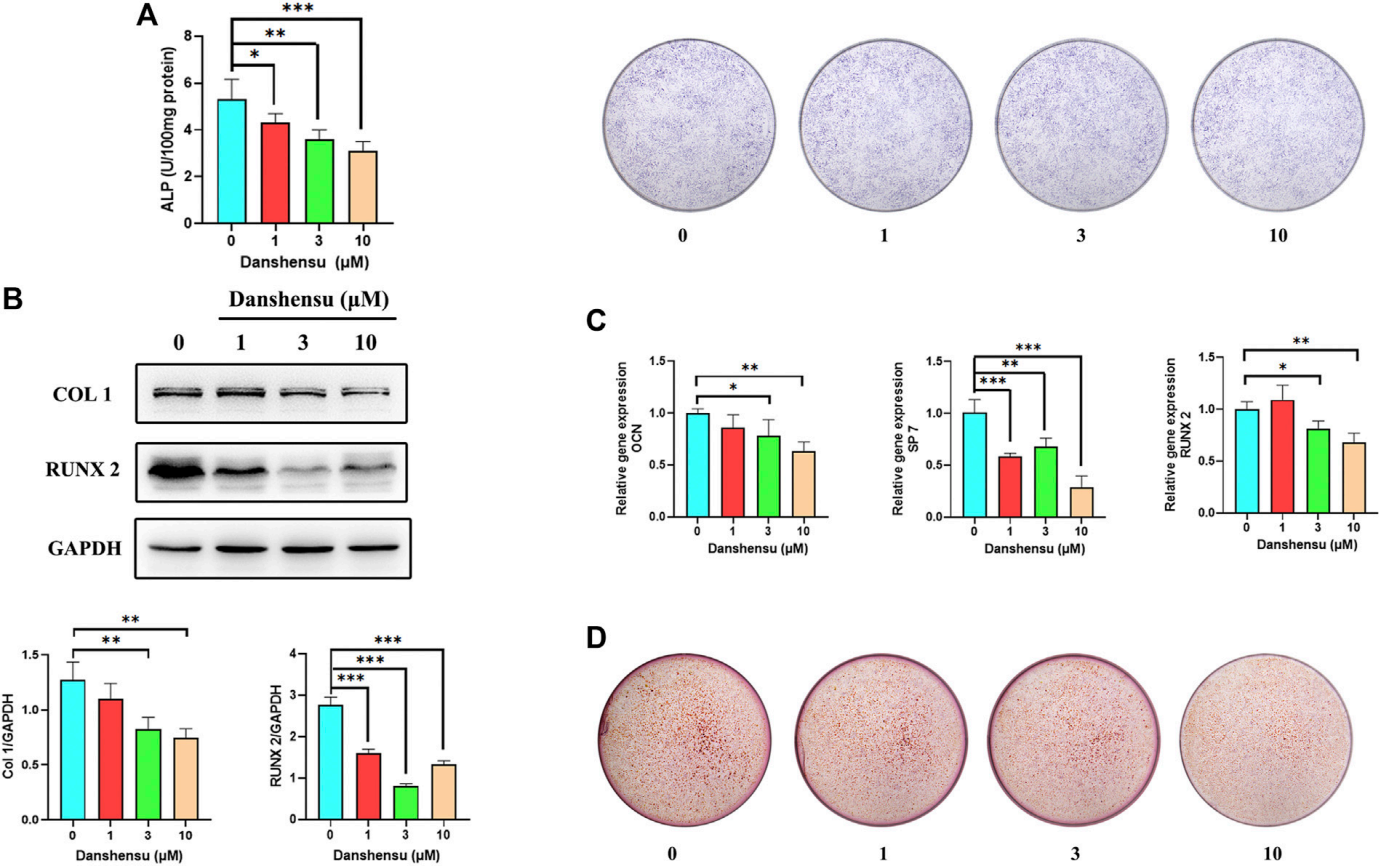
Danshensu suppresses the ossification of AS fibroblasts. **(A)** ALP activity and staining of AS fibroblasts treated for 7 days with different concentrations of Danshensu. **(B)** RUNX2 and COL1 protein levels on day 14. **(C)** SP7, OCN, and RUNX2 mRNA expression in fibroblasts on day 14. **(D)** ARS of osteoblasts on day 30. *p < 0.05, **p < 0.01, ***p < 0.001 vs. 0 group, *n* = 3.

As fibroblasts are known to contribute to heterotopic ossification, we extracted primary fibroblasts from hip ligaments of AS patients and treated these cells with Danshensu (1, 3, and 10 μM) to further assess whether Danshensu inhibits ectopic ossification in AS. The ALP staining and activity assay showed that Danshensu suppressed osteogenic differentiation of AS fibroblasts (Figure 9A). This was accompanied by decreased expression of COL1 and RUNX2 proteins (Figure 9B) and downregulation of OCN, SP7, and RUNX2 transcripts (Figure 9C) as determined by western blotting and qPCR, respectively. ARS confirmed the results of ALP staining (Figure 9D).

### Danshensu Suppresses JNK and ERK Phosphorylation in AS Fibroblasts

**FIGURE 10:**
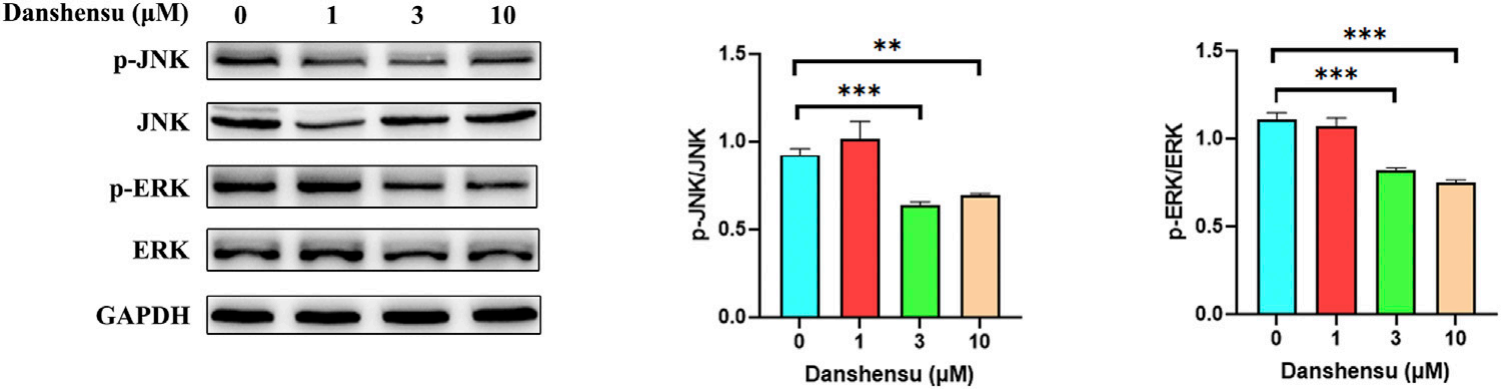
Danshensu suppresses JNK and ERK phosphorylation in AS fibroblasts. p-JNK and p-ERK protein levels in AS fibroblasts treated with Danshensu for 14 days. **p < 0.01, ***p < 0.001 vs. 0 group, *n* = 3.

Based on the results of the network pharmacology analysis, we examined the phosphorylation levels of JNK and ERK in AS fibroblasts treated with Danshensu for 14 days. The results showed that p-JNK and p-ERK levels were significantly downregulated in cells treated with 3 and 10 μM Danshensu (Figure 10).

## Discussion

AS is a chronic inflammatory disease with complex pathophysiology and no effective treatments (Magrey and Khan, 2017). TNF-α and IL-17A play important roles in the development of AS (Wang et al., 2020). Although TNF-α blocker and IL-17A inhibitor are used for AS treatment, the side effects and high cost limit their widespread use. Traditional Chinese medicine is known for its multitarget, bidirectional, and systemic regulatory effects with fewer side effects but has not been applied to AS. Danshensu is a pure molecule with a clearly defined structure that is extracted from the widely used traditional Chinese medicine Danshen (Zhang et al., 2019). This study provides the first evidence for the anti-osteogenic effect of Danshensu in AS based on in silico and *in vitro* data.

Network pharmacology allows visualization of the relationship between biological processes and drug action through construction of an interaction network (Gao et al., 2020). The mechanism of action of drugs can be explained by analysis of network components including drug targets, diseases, and molecular pathways (Li et al., 2020). It is particularly suitable for the study of multicomponent, multitarget, and multi-mechanism traditional Chinese medicines. In this study, we used a network pharmacology-based approach to identify targets, biological processes, and signaling pathways of Danshensu in AS. Danshensu target genes shared by AS were mostly related to the inflammatory response, cell proliferation and differentiation, fibrosis, and arthritis. The top gene, *IL6*, is known to promote AS fibroblast ossification *via* MAPK/ERK signaling (Li et al., 2016). The results of the GO functional annotation and KEGG pathway enrichment analysis showed that the therapeutic effect of Danshensu may be related to the regulation of inflammatory response; protein transport; oxidative stress; cell apoptosis; and ERK, JNK, and MAPK signaling. The disease–drug component–target–signaling pathway interaction network revealed that the JNK and ERK pathways were closely related to the 10 hub genes, suggesting that they play a critical role in the anti-osteogenic effect of Danshensu. The ERK and MAPK pathways were also related to most AS targets of Danshensu. The MAPK pathway including ERK, JNK and p38 pathways, is involved in inflammatory signaling, regulation of cell proliferation and differentiation, and activation of the immune system (Lai et al., 2020). The ERK1/2, JNK, and p38 pathways regulate osteoblast differentiation (Chen et al., 2016), and inhibition of MAPK signaling was shown to reduce the expression levels of ALP and OCN (Wang et al., 2019). JNK is a downstream effector of the proinflammatory cytokine-induced immune response in AS. Additionally, Wnt gene promoters contain p65 and c-Jun binding sites, and inhibiting the NF-κB and JNK/AP-1 pathways suppressed the TNF-α–induced upregulation of Wnt expression (Li et al., 2018) and attenuated IL-17–induced inflammatory injury (Mao et al., 2019) in AS.

We confirmed the anti-osteogenic effect of Danshensu in AS using an *in vitro* model established by treating osteoblasts with low concentrations of TNF-α and AS fibroblasts. Fibroblasts and osteoblasts originate from mesenchymal stem cells and share a similar phenotype and differentiation pathway that makes their reciprocal transformation possible (Zou et al., 2016). AS fibroblasts have higher osteogenic potential than healthy control fibroblasts and may undergo ectopic ossification (Qin et al., 2018; Zhang et al., 2021). Consistent with the previous finding that mild inflammation promotes new bone formation of AS (Li et al., 2018), we confirmed in the present study that treatment with a low concentration dose of TNF-α promoted osteoblast differentiation in normal osteoblasts. We therefore used 0.01 ng/ml TNF-α to mimic the AS inflammatory microenvironment *in vitro*. Our results showed that Danshensu prevented the ossification of TNF-α–treated osteoblasts in a dose-dependent manner. Notably, osteogenic activity markers were upregulated in osteoblasts exposed to Danshensu alone. These results indicate that Danshensu inhibits osteogenesis in osteoblasts exposed to low-intensity inflammatory stimulation while preserving their osteogenic potential. This effect of Danshensu is likely related to its regulation of inflammatory response as revealed in network pharmacology analysis. Importantly, Danshensu also suppressed osteogenic differentiation in AS fibroblasts, demonstrating that Danshensu can prevent abnormal new bone formation in AS.

The results of the GO and KEGG pathway enrichment analyses along with the target–signaling pathway interaction network indicated that JNK and ERK signaling pathways are involved in the anti-osteogenic effect of Danshensu in AS. We found that Danshensu treatment decreased the phosphorylation of JNK and ERK in AS fibroblasts, suggesting that Danshensu prevents ectopic ossification in AS *via* inhibition of these pathways. Interestingly, we previously found that salvianolic acid B, another bioactive component of Danshen, promoted osteogenesis of normal hMSCs by increasing ERK1/2 signaling (Xu et al., 2014). The opposite effects of Danshensu on normal vs abnormal cells may be related to the distinct microenvironments of the two cell types. The immune microenvironment in AS is characterized by activated inflammatory mediators that promote new bone formation (Schett et al., 2017). The proinflammatory cytokine TNF-α at a low concentration may exert this effect, which is countered by Danshensu *via* modulation of JNK and ERK signaling pathways. However, the detailed regulatory mechanism of Danshensu in the immune microenvironment in AS remains to be elucidated.

Besides JNK and ERK, other pathways that were found to be enriched in our analyses may be involved in the molecular mechanism of Danshensu in AS such as phosphatidylinositol 3-kinase (PI3K)–protein kinase B (AKT) and Janus kinase (JAK)–signal transducer and activator of transcription (STAT). PI3K–AKT signaling promotes the proliferation and differentiation of osteoblast precursors while p38 MAPK signaling regulates the proliferation and differentiation of osteoclast progenitor cells (Han and Choi, 2017; Bai et al., 2018). The JAK–STAT pathway affects bone formation and the mechanical strength of bone (Sanpaolo et al., 2020). Forkhead box (Fox)O was shown to be associated with the regulation of PI3K–AKT signaling in mechanically stimulated osteoblasts (Li et al., 2010). Activation of hypoxia-inducible factor (HIF) along with an increased supply of oxygen and nutrients was proposed to underlie bone anabolism in mice lacking the oxygen sensor prolyl hydroxylase (PHD)2 (Stegen et al., 2018). Additionally, the enrichment of the apoptosis cascade and calcium signaling pathway in our KEGG analysis suggests that Danshensu also exerts an anti-osteogenic effect in AS by inducing apoptosis in osteoblasts and promoting the uptake of external calcium ions by the body.

In conclusion, we identified potential targets of Danshensu in AS and showed that Danshensu suppresses the osteogenic differentiation of osteoblasts and AS fibroblasts *via* activation of JNK and ERK signaling pathways. Although the putative targets of Danshensu in AS need to be validated by *in vivo* studies, our results provide molecular-level evidence for the therapeutic potential of Danshensu in the treatment of AS.

## Data Availability

The original contributions presented in the study are included in the article/Supplementary Material, further inquiries can be directed to the corresponding authors.

## References

[B1] Bai B. L. Xie Z. J. Weng S. J. Wu Z. Y. Li H. Tao Z. S. (2018). Chitosan Oligosaccharide Promotes Osteoclast Formation by Stimulating the Activation of MAPK and AKT Signaling Pathways. J. Biomater. Sci. Polym. Ed. 29 (10), 1207–1218. 10.1080/09205063.2018.1448336 29502489

[B2] Chen D. Liu Y. Yang H. Chen D. Zhang X. Fermandes J. C. (2016). Connexin 43 Promotes Ossification of the Posterior Longitudinal Ligament through Activation of the ERK1/2 and P38 MAPK Pathways. Cell Tissue Res 363 (3), 765–773. 10.1007/s00441-015-2277-6 26334722

[B3] Frost A. Jonsson K. B. Nilsson O. Ljunggren O. (1997). Inflammatory Cytokines Regulate Proliferation of Cultured Human Osteoblasts. Acta Orthop. Scand. 68 (2), 91–96. 10.3109/17453679709003987 9174441

[B4] Gao K. Song Y. P. Song A. (2020). Exploring Active Ingredients and Function Mechanisms of Ephedra-Bitter almond for Prevention and Treatment of Corona Virus Disease 2019 (COVID-19) Based on Network Pharmacology. BioData Min 13 (1), 19. 10.1186/s13040-020-00229-4 33292385 PMC7653455

[B5] Han I. Choi E. H. (2017). The Role of Non-thermal Atmospheric Pressure Biocompatible Plasma in the Differentiation of Osteoblastic Precursor Cells, MC3T3-E1. Oncotarget 8 (22), 36399–36409. 10.18632/oncotarget.16821 28432281 PMC5482663

[B6] Lai B. Wu C. H. Lai J. H. (2020). Activation of C-Jun N-Terminal Kinase, a Potential Therapeutic Target in Autoimmune Arthritis. Cells 9 (11), 2466. 10.3390/cells9112466 PMC7696795 33198301

[B7] Li C. J. Chang J. K. Chou C. H. Wang G. J. Ho M. L. (2010). The PI3K/Akt/FOXO3a/p27Kip1 Signaling Contributes to Anti-inflammatory Drug-Suppressed Proliferation of Human Osteoblasts. Biochem. Pharmacol. 79 (6), 926–937. 10.1016/j.bcp.2009.10.019 19883628

[B8] Li D. H. He C. R. Liu F. P. Li J. Gao J. W. Li Y. (2016). Annexin A2, Up-Regulated by IL-6, Promotes the Ossification of Ligament Fibroblasts from Ankylosing Spondylitis Patients. Biomed. Pharmacother. 84, 674–679. 10.1016/j.biopha.2016.09.091 27697640

[B9] Li X. Wang J. Zhan Z. Li S. Zheng Z. Wang T. (2018). Inflammation Intensity-dependent Expression of Osteoinductive Wnt Proteins Is Critical for Ectopic New Bone Formation in Ankylosing Spondylitis. Arthritis Rheumatol. 70 (7), 1056–1070. 10.1002/art.40468 29481736

[B10] Li Y. Q. Chen Y. Fang J. Y. Jiang S. Q. Li P. Li F. (2020). Integrated Network Pharmacology and Zebrafish Model to Investigate Dual-Effects Components of Cistanche Tubulosa for Treating Both Osteoporosis and Alzheimer's Disease. J. Ethnopharmacol 254, 112764. 10.1016/j.jep.2020.112764 32173426

[B11] Liu L. Yuan Y. Zhang S. Xu J. Zou J. (2021). Osteoimmunological Insights into the Pathogenesis of Ankylosing Spondylitis. J. Cel Physiol 236 (9), 6090–6100. 10.1002/jcp.30313 33559242

[B12] Lories R. J. (2018). Advances in Understanding the Pathophysiology of Spondyloarthritis. Best Pract. Res. Clin. Rheumatol. 32 (3), 331–341. 10.1016/j.berh.2018.12.001 31171306

[B13] Magrey M. N. Khan M. A. (2017). The Paradox of Bone Formation and Bone Loss in Ankylosing Spondylitis: Evolving New Concepts of Bone Formation and Future Trends in Management. Curr. Rheumatol. Rep. 19 (4), 17. 10.1007/s11926-017-0644-x 28361330

[B14] Maksymowych W. P. Chiowchanwisawakit P. Clare T. Pedersen S. J. Østergaard M. Lambert R. G. (2009). Inflammatory Lesions of the Spine on Magnetic Resonance Imaging Predict the Development of New Syndesmophytes in Ankylosing Spondylitis: Evidence of a Relationship between Inflammation and New Bone Formation. Arthritis Rheum. 60 (1), 93–102. 10.1002/art.24132 19116919

[B15] Mao D. Li H. Zhang L. Xu J. Yu C. Zhang Q. (2019). Bilobalide Alleviates IL-17-induced Inflammatory Injury in ATDC5 Cells by Downregulation of microRNA-125a. J. Biochem. Mol. Toxicol. 33 (12), e22405. 10.1002/jbt.22405 31593333

[B16] Meng Y. Li W. Z. Shi Y. W. Zhou B. F. Ma R. Li W. P. (2016). Danshensu Protects against Ischemia/reperfusion Injury and Inhibits the Apoptosis of H9c2 Cells by Reducing the Calcium Overload through the P-JNK-NF-Κb-TRPC6 Pathway. Int. J. Mol. Med. 37 (1), 258–266. 10.3892/ijmm.2015.2419 26718129

[B17] Pedersen S. J. Sørensen I. J. Lambert R. G. Hermann K. G. Garnero P. Johansen J. S. (2011). Radiographic Progression Is Associated with Resolution of Systemic Inflammation in Patients with Axial Spondylarthritis Treated with Tumor Necrosis Factor α Inhibitors: a Study of Radiographic Progression, Inflammation on Magnetic Resonance Imaging, and Circulating Biomarkers of Inflammation, Angiogenesis, and Cartilage and Bone Turnover. Arthritis Rheum. 63 (12), 3789–3800. 10.1002/art.30627 22127697

[B18] Poddubnyy D. Sieper J. (2017). Mechanism of New Bone Formation in Axial Spondyloarthritis. Curr. Rheumatol. Rep. 19 (9), 55. 10.1007/s11926-017-0681-5 28752489

[B19] Poddubnyy D. Sieper J. (2020). Treatment of Axial Spondyloarthritis: What Does the Future Hold? Curr. Rheumatol. Rep. 22 (9), 47. 10.1007/s11926-020-00924-5 32691259 PMC7371669

[B20] Qin X. Jiang T. Liu S. Tan J. Wu H. Zheng L. (2018). Effect of Metformin on Ossification and Inflammation of Fibroblasts in Ankylosing Spondylitis: An *In Vitro* Study. J. Cel Biochem 119 (1), 1074–1082. 10.1002/jcb.26275 28696014

[B21] Sanpaolo E. R. Rotondo C. Cici D. Corrado A. Cantatore F. P. (2020). JAK/STAT Pathway and Molecular Mechanism in Bone Remodeling. Mol. Biol. Rep. 47 (11), 9087–9096. 10.1007/s11033-020-05910-9 33099760 PMC7674338

[B22] Schett G. Lories R. J. D'Agostino M. A. Elewaut D. Kirkham B. Soriano E. R. (2017). Enthesitis: from Pathophysiology to Treatment. Nat. Rev. Rheumatol. 13 (12), 731–741. 10.1038/nrrheum.2017.188 29158573

[B23] Stegen S. Stockmans I. Moermans K. Thienpont B. Maxwell P. H. Carmeliet P. (2018). Osteocytic Oxygen Sensing Controls Bone Mass through Epigenetic Regulation of Sclerostin. Nat. Commun. 9 (1), 2557. 10.1038/s41467-018-04679-7 29967369 PMC6028485

[B24] Van Mechelen M. Gulino G. R. de Vlam K. Lories R. (2018). Bone Disease in Axial Spondyloarthritis. Calcif Tissue Int. 102 (5), 547–558. 10.1007/s00223-017-0356-2 29090349

[B25] van Tok M. N. van Duivenvoorde L. M. Kramer I. Ingold P. Pfister S. Roth L. (2019). Interleukin-17A Inhibition Diminishes Inflammation and New Bone Formation in Experimental Spondyloarthritis. Arthritis Rheumatol. 71 (4), 612–625. 10.1002/art.40770 30390386

[B26] Wang H. Zheng H. Ma Y. (2020). Drug Treatment of Ankylosing Spondylitis and Related Complications: an Overlook Review. Ann. Palliat. Med. 9 (4), 2279–2285. 10.21037/apm-20-277 32576001

[B27] Wang Q. Yang Q. Zhang A. Kang Z. Wang Y. Zhang Z. (2019). Silencing of SPARC Represses Heterotopic Ossification *via* Inhibition of the MAPK Signaling Pathway. Biosci. Rep. 39 (11), BSR20191805. 10.1042/bsr20191805 31548362 PMC6851515

[B28] Wang W. Li S. S. Xu X. F. Yang C. Niu X. G. Yin S. X. (2021). Danshensu Alleviates Pseudo-typed SARS-CoV-2 Induced Mouse Acute Lung Inflammation. Acta Pharmacol. Sin, 1–10. 10.1038/s41401-021-00714-4 34267343 PMC8280584

[B29] Xie C. Luo J. Hu H. Wang L. Yu P. Xu L. (2021). A Novel Danshensu/tetramethypyrazine Derivative Attenuates Oxidative Stress-Induced Autophagy Injury *via* the AMPK-mTOR-Ulk1 Signaling Pathway in Cardiomyocytes. Exp. Ther. Med. 21 (2), 118. 10.3892/etm.2020.9550 33335581 PMC7739857

[B30] Xu D. Xu L. Zhou C. Lee W. Y. Wu T. Cui L. (2014). Salvianolic Acid B Promotes Osteogenesis of Human Mesenchymal Stem Cells through Activating ERK Signaling Pathway. Int. J. Biochem. Cel Biol 51, 1–9. 10.1016/j.biocel.2014.03.005 24657587

[B31] Yan X. Wu H. Wu Z. Hua F. Liang D. Sun H. (2017). The New Synthetic H2S-Releasing SDSS Protects MC3T3-E1 Osteoblasts against H2O2-Induced Apoptosis by Suppressing Oxidative Stress, Inhibiting MAPKs, and Activating the PI3K/Akt Pathway. Front. Pharmacol. 8, 07. 10.3389/fphar.2017.00007 28163684 PMC5247634

[B32] Yin Y. Wang M. Liu M. Zhou E. Ren T. Chang X. (2020). Efficacy and Safety of IL-17 Inhibitors for the Treatment of Ankylosing Spondylitis: a Systematic Review and Meta-Analysis. Arthritis Res. Ther. 22 (1), 111. 10.1186/s13075-020-02208-w 32398096 PMC7216398

[B33] Zeng H. Su S. Xiang X. Sha X. Zhu Z. Wang Y. (2017). Comparative Analysis of the Major Chemical Constituents in Salvia Miltiorrhiza Roots, Stems, Leaves and Flowers during Different Growth Periods by UPLC-TQ-MS/MS and HPLC-ELSD Methods. Molecules 22 (5), 771. 10.3390/molecules22050771 PMC6154317 28489029

[B34] Zhang J. Zhang Q. Liu G. Zhang N. (2019). Therapeutic Potentials and Mechanisms of the Chinese Traditional Medicine Danshensu. Eur. J. Pharmacol. 864, 172710. 10.1016/j.ejphar.2019.172710 31586468

[B35] Zhang Y. Chen W. G. Yang S. Z. Qiu H. Hu X. Qiu Y. Y. (2021). Up-regulation of TβRIII Facilitates the Osteogenesis of Supraspinous Ligament-Derived Fibroblasts from Patients with Ankylosing Spondylitis. J. Cel Mol Med 25 (3), 1613–1623. 10.1111/jcmm.16262 PMC7875912 33410269

[B36] Zou Y. C. Yan L. M. Gao Y. P. Wang Z. Y. Liu G. (2020). miR-21 May Act as a Potential Mediator between Inflammation and Abnormal Bone Formation in Ankylosing Spondylitis Based on TNF-α Concentration-dependent Manner through the JAK2/STAT3 Pathway. Dose Response 18 (1), 1559325819901239. 10.1177/1559325819901239 32009856 PMC6974759

[B37] Zou Y. C. Yang X. W. Yuan S. G. Zhang P. Li Y. K. (2016). Celastrol Inhibits Prostaglandin E2-Induced Proliferation and Osteogenic Differentiation of Fibroblasts Isolated from Ankylosing Spondylitis Hip Tissues *In Vitro* . Drug Des. Devel Ther. 10, 933–948. 10.2147/dddt.S97463 PMC4790082 27022241

